# Expression Levels and Clinical Values of miR-195, miR-424, miR-10b, miR-103a-3p, and miR-542-3p in Vasculo-Behçet’s Disease

**DOI:** 10.31138/mjr.030623.elc

**Published:** 2023-09-07

**Authors:** Serdar Kaymaz, Demiray Aydın, Karasu Uğur, Veli Çobankara, Seçil Tan

**Affiliations:** 1 Department of Rheumatology;; 2 Department of Genetics;; 3 Department of Cancer Molecular Biology, Pamukkale University, Denizli, Turkey

**Keywords:** Behçet’s disease, microRNA-195, serum, real-time quantitative PCR

## Abstract

**Objective:**

MicroRNAs (miRNAs) are involved in a range of pathological and biological processes. Vascular involvement is an important complication associated with morbidity and mortality in Behçet’s disease (BD). In this study, we aimed to evaluate the expression levels of miR-195, miR-424, miR-10b, miR-103a-3p, and miR-542-3p in Turkish patients with BD, and their possible association with vascular involvement and clinical activity.

**Methods:**

This cross-sectional study included 61 BD patients and 25 age- and sex-matched healthy individuals. The patients were categorised into two groups based on the presence or absence of vascular involvement. Demographic data, disease duration, disease activity, and medical treatments were recorded. Disease activity was evaluated using the Behçet’s Disease Current Activity Form (BDCAF) and the Behçet’s Syndrome Activity Scale (BSAS). The expression levels of miRNAs were measured using real-time quantitative polymerase chain reaction (RT-qPCR).

**Results:**

The comparison of the clinical features of BD patients with and without vascular involvement revealed no significant difference. However, the expression levels of miR-195, miR-424, miR-10b, miR-103a-3p, and miR-542-3p were significantly higher in BD patients than in healthy controls (p<0.001, p<0.001, p=0.010, p<0.01, p=0.039, respectively). Moreover, the expression level of miR-195 was significantly higher in vasculo-Behçet patients than in the other groups (p=0.0318). However, no significant association was found between the expression levels of miR-195 and clinical activity.

**Conclusion:**

Our study results indicated elevated serum levels of miR-195 in BD patients, which may be associated with vascular involvement. Therefore, miR-195 could potentially serve as a biomarker for the diagnosis and monitoring of vasculo-Behçet’s disease.

## INTRODUCTION

Behçet’s disease (BD) is an inflammatory condition of unknown origin, which has a higher prevalence in countries along the ancient Silk Road.^1^ The development mechanism of BD is not yet understood, but abnormal immune activity triggered by environmental sources or certain stimuli such as infection may play a crucial role.^2^ BD can manifest with a variety of systemic symptoms, including recurrent oral aphthous ulcers, arthritis, skin lesions, gastrointestinal involvement, and neurological complications.^1^ However, BD can also cause vascular complications associated with morbidity and mortality.^3^ The incidence of vascular involvement in BD is reported to be between 6.3–15.3%.^4,5^ Current research suggests that the manifestations of vascular BD have inflammatory characteristics, which are associated with endothelial dysfunction, oxidative modifications of coagulation proteins, and enhanced adhesiveness.^6–8^ Furthermore, BD vasculitis is characterised by a high level of hypercoagulability, leading to excessive thrombin production, decreased fibrinolysis, platelet hyperactivity, and the formation of platelet/neutrophil complexes.^9,10^ These changes can contribute to the formation of thrombosis.

MicroRNAs (miRNAs) are small noncoding RNAs that regulate the degradation or translation inhibition of target mRNAs by base pairing with certain regions of the mRNA.^11^ By controlling the expression of multiple mRNA targets, miRNAs epigenetically modulate thirty percent of human genes. As a result, they provide insight into the functions of pathogenetic pathways and information on the pathophysiological processes of various human diseases.^12,13^ Given these properties, miRNAs have potential roles in a diverse range of human diseases, including immune-mediated and vascular conditions. In particular, miRNAs regulate cytokines that play a critical role in the development of BD.^14–17^ For instance, Chen et al. reported that the increased expression of miRNAs is associated with the upregulation of tumour necrosis factor-alpha (TNF-α) and the downregulation of the cytotoxic T lymphocyte-associated antigen-4 (CTLA-4) genes in BD patients.^14^ Additionally, Abdelaleem et al. found that miR-146a (rs57095329) is associated with BD, and certain alleles and genotypes are linked to neurological manifestations and ocular and oral ulcers, but not with vascular disease.^16^

Recent research has indicated that several miRNAs, including miR-195, miR-424, miR-10b, miR-103a-3p, and miR-542-3p, are associated with vascular complications. In a study by Cheng et al., miR-195 was linked to the downregulation of adhesion molecules and the upregulation of endothelial nitric oxide synthase expression, along with inhibition of nuclear factor-kappa B (NF-kB), which can affect the expression of adhesion and inflammatory molecules in endothelial cells.^18^ Meanwhile, according to Liu et al., miR-424 may play a critical role in suppressing endothelial differentiation induced by basic fibroblast growth factor and vascular endothelial growth factor.^19^ Yu et al. found that miR-10b stimulates vascular muscle cell proliferation through the Akt pathway by targeting the Tat-interacting protein 30.^20^ Moreover, miR-103a-3p may downregulate the expression of chemokine C-X-C motif ligand 12 (CXCL12) to interrupt thrombosis and inflammatory response, which can prevent venous thrombosis.^21^ Lastly, miR-542-3p has been linked to vessel wall calcification and the osteogenic transition of VSM cells.^22^ Despite these findings, there is a lack of information on the serum levels of these miRNAs in vasculo-Behçet’s disease. Therefore, this study aimed to evaluate the association of miR-195, miR-424, miR-10b, miR-103a-3p, and miR-542-3p with vascular involvement and clinical activity in BD patients.

## MATERIALS AND METHODS

This cross-sectional study was conducted at the Department of Rheumatology of a university hospital between January 2020 and April 2021. The sample size was determined using G*Power version 3.1.7 (University of Kiel, Kiel, Germany). Based on the research results of Hong et al., it was determined that 12 subjects per group were needed to achieve 95% power, assuming an expected average value of 2.1 in the first group (with a standard deviation of 0.81) and 3.53 (with a standard deviation of 1.19) in the second group.^23^

A detailed medical history was obtained from all participants, and physical examinations were performed. The study included 66 BD patients (40 males, 26 females; mean age: 40.5 ± 11.2 years) who fulfilled the International Criteria for Behçet’s Disease, as well as 25 age-, body mass index (BMI) and gender-matched healthy individuals (16 males, 9 females; mean age: 39.7 ± 9.9 years) who did not have any signs or symptoms suggestive of medical comorbidity.^24^

All patients with BD underwent a detailed medical history and physical examination. Routine laboratory tests, including fasting blood glucose, complete blood count, and liver and kidney function tests, were performed and found to be within normal limits for all participants.

BD patients were categorised into two groups based on the presence or absence of vascular involvement. Vascular involvement was diagnosed when a lesion in the aorta, small artery, or large or small vessels was confirmed clinically and radiologically. The specific patterns of vascular involvement were determined according to established criteria.^25^ Patients with a history of thrombophilia, antiphospholipid syndrome, systemic vasculitis, hematologic disorders, autoimmune diseases, hyper-lipidaemia, diabetes mellitus, malignancy, heart failure, or terminal conditions such as end-stage renal disease were excluded from the study.

Disease activity was assessed using two different scales: the Behçet’s Syndrome Activity Scale (BSAS) and the Behçet’s Disease Current Activity Form (BDCAF).^25–27^ In the BSAS scale, the patient rates the level of discomfort due to current disease activity, genital ulcers, oral ulcers, and skin lesions using visual analog scales.^26^ On the other hand, the BDCAF scale takes into account the presence of new central nervous system involvement and new vascular involvement within the four weeks preceding the visit when evaluating oral ulcer, genital ulcer, erythema nodosum, pustule, arthralgia, arthritis, diarrhoea, nausea/vomiting, headache, and eye inflammation.

### RNA extraction

To isolate total RNA from serum samples, 5 ml blood samples were collected from both the control and patient groups and centrifuged at 4000 rpm for 10 minutes. The QiagenmiRNeasy kit (catalogue no:217184, Germany) protocol was used for total RNA isolation. According to the protocol, 900 μl of QIAzol was added to 100 μl of serum sample and incubated for 5 minutes at room temperature. Then, 180 μl of chloroform was added and the solution was vortexed for 15 seconds. The solution was then centrifuged at 12000 xg for 15 minutes at +4 °C, and the top clear phase was carefully transferred to a new tube. Ethanol absolute 96% was added, 1.5 times the total volume, and the solution was centrifuged at 8000 xg for 30 seconds to flow through the columns included in the kit. The column was moved to a new tube and washed by centrifuging 500 μl of RWT solution included in the kit at 8000 xg for 30 seconds. The same process was repeated with RPM solutions. Next, 500 μl of 80% ethanol was added and centrifuged at 8000 xg for 2 minutes, followed by centrifugation at 8000 xg for 4 minutes to remove the ethanol. Finally, RNase-free water was added, and the mixture was centrifuged at 10000 xg for 1 minute to obtain cell-free total RNA. The obtained RNA was measured using the NanoDrop and stored at −80 °C.

#### cDNA Synthesis

The isolated RNAs were converted into cDNA following the miRNA All-In-One cDNA Synthesis Kit (Cat. No. G898 ABM, Canada) protocol. To prepare the reaction mixture, 10μl of 2X miRNA cDNA Synthesis SuperMix, 2μl of total RNA, 2μl of Enzyme Mix, and 4μl of RNAse-free water were added. The reaction conditions were as follows: incubation at 37 °C for 30 minutes, at 50 °C for 15 minutes, and then at 85 °C for 5 minutes to immediately terminate the reaction. Finally, the samples were stored at −20 °C.

#### Quantitative PCR

Mature miRNA expression was measured using TaqMan® Advanced miRNA assay chemistry. For the real-time PCR reaction, BlasTaq™ 2X PCR MasterMix from ABM (Canada) was used along with hsa-miR-542-3p, hsa-miR-424, hsa-miR-10b, hsa-miR-195, and hsamiR-103a-3p primers and the cDNAs obtained from the samples. The reaction mixture was prepared as follows: 10 μl of master mix, 0.5 μl of universal primer, 0.5 μl of primer, 8 μl of RNase-free water, and 1 μl of cDNA, with a total volume of 20 μl. The reaction conditions were as follows: one step at 95 °C for 5 minutes, followed by 40 steps at 95 °C for 15 seconds, at 62 °C for 1 minute, and at 72 °C for 1 minute. The expressions of all samples were calculated against the miR-39 spike using the delta-delta Ct (2−ΔΔCt) formula.

### Statistical Analysis

The statistical analyses were conducted using GraphPad Prism version 8.0.2 software. Descriptive statistics were used to present demographic characteristics. The normality and non-normality distribution of variables were determined using the Kolmogorov-Smirnov and non-parametric tests, respectively. Continuous variables were presented in median and interquartile range (IQR), while categorical variables were presented in number and percentage. The Kruskal-Wallis test was employed to analyse significant differences in continuous variables, while the Chi-Square test was used for categorical variables. Inter-group comparisons were made using the Kruskal-Wallis test and the post hoc Bonferroni correction (Mann-Whitney U test). Spearman’s correlation analysis was used to evaluate the correlation between non-parametric variables. A correlation coefficient (r) of less than 0.2 was considered negligible, 0.2 to 0.4 as fair, 0.41 to 0.60 as moderate, 0.61 to 0.80 as good, and 0.8 as excellent. A p-value of less than 0.05 indicated statistical significance.

## RESULTS

**Figure 1.**
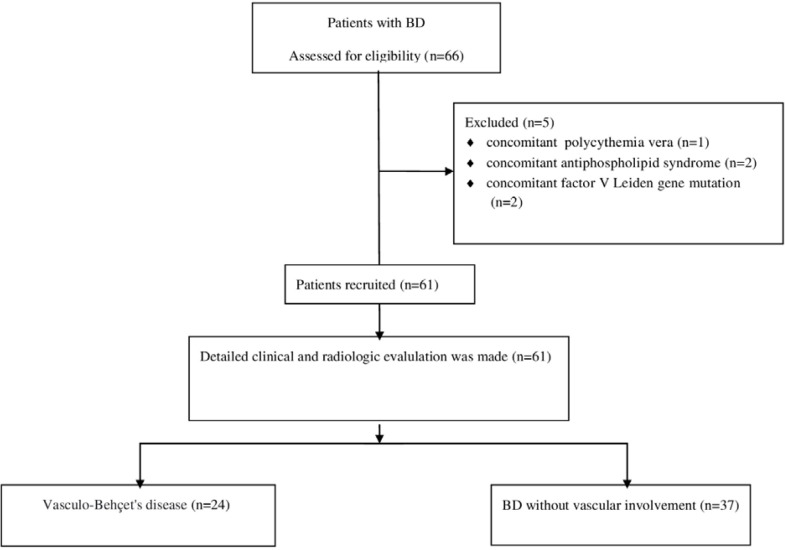
Flowchart of patients recruited for the study.

Sixty-six patients were enrolled in the study; however, 5 patients were excluded for not meeting the inclusion criteria. Among the included patients, 24 had vascular involvement and were classified as Group 1, while 37 did not have vascular involvement and were classified as Group 2 (as shown in **Figure 1**). There were no significant differences in clinical characteristics between the two groups (as shown in **Table 1**).

**Table 1.** Demographic and clinical characteristics of the groups and laboratory characteristics of participants and medications.

	**Group 1 (n=24) BD with vascular involvement**	**Group 2 (n=37) BD without vascular involvement**	**Group 3 (n=33) Healthy controls**	*p* **-value**
Gender, n (%)				
*-Male*	16 (67)	22 (59)	14 (42)	.179
*-Female*	8 (33)	15 (41)	19 (58)	
Age (year), Median (Min-Max), 95% CI	37 (26-72), (35.8-45.4)	43 (18-72), (37.1-44.6)	41 (21-67), (36.8-44.2)	.992
Disease duration (year), Median (Min-Max), 95% CI	7 (1-22), (5.6-11.1)	8 (1-24), (7.1-10.6)	-	.758
Medical Treatment, n (%)				
-Colchicine	3 (12.3)	10 (27)		.090
-Immunosuppressive agents	20 (82.8)	21 (56.8)		
-Interferon	1 (4.1)	6 (16.2)		
-Anticoagulant	12 (50)	-		
BD manifestations, n (%)				
-Mucocutaneous	11 (45.8)	17 (45.9)	-	.970
-Ocular + Mucocutaneous	10 (41.6)	14 (37.8)		-
-Articular + Mucocutaneous	1 (1.4)	2 (5.4)		-
-Neurologic + Mucocutaneous	2 (2.8)	4 (10.8)		-
Disease activation, Median (Min-Max), 95% CI				
-BDCAF	2 (0-8), (1.5-3.5)	1 (0-7), (1.1-2.5)	-	.494
-BSAS	1.7 (0-9.5), (1.2-3.2)	1 (0-9), (1.1-2.5)	-	.778
Vascular manifestation, n (%)				
*-DVT*	12 (50)	-		
*-Portal vein thrombosis*	1 (4.1)	-		
*-Cerebral sinus vein thrombosis*	3 (12.3)	-		
*-Retinal vein occlusion*	3 (12.3)	-		
*-Pulmonary embolism*	2 (8.2)	-		
*-Abdominal aortic aneurysm*	1 (4.1)	-		
*-Jugular vein thrombosis*	2 (8.2)	-		

Chi-Square, Kruskal-Wallis, and Mann-Whitney U tests were used.

* -p<0.05: statistically significant; BD: Behcet's Disease; BDCAF: Behcet's Disease Current Activity Form; BSAS: Behcet's Syndrome Activity Scale; DVT: Deep Vein Thrombosis; SD: Standard Deviation; Min: Minimum; Max: Maximum; CI: Confidence interval.

**Figure 2.**
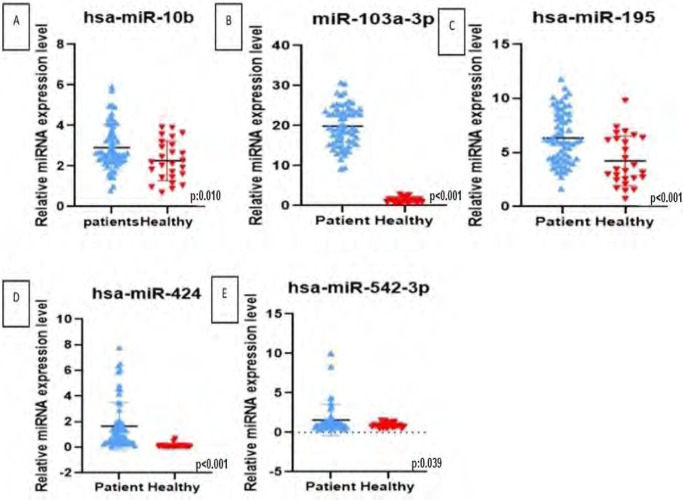
Expression levels of miR-10b, miR-103a-3p, miR-195, miR-424, and miR-542-3p in patients with Behçet’s disease and healthy controls (**A–E**).

**Figure 3.**
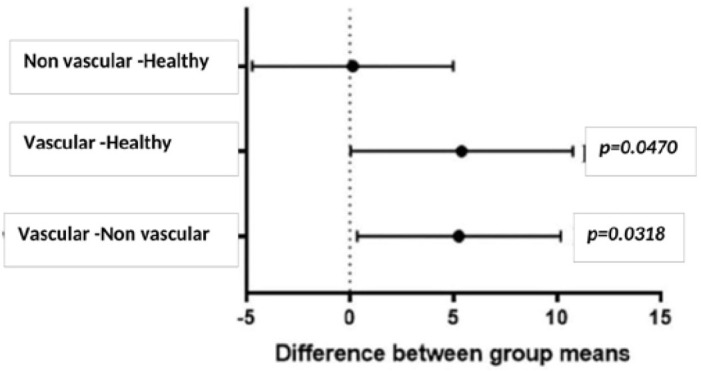
Different expression levels of miR-195 between the groups.

Compared to healthy controls, BD patients had significantly different expression levels of miR-195, miR-424, miR-10b, miR-103a-3p, and miR-542-3p (p<0.001, p<0.001, p=0.010, p<0.001, p=0.039, respectively) (as shown in **Figure 2**). Interestingly, Group 1 had significantly higher serum levels of miR-195 expression compared to both Group 2 and Group 3 (p=0.0318, p=0.047, respectively) (as shown in **Figure 3**).

There was no significant correlation between the serum levels of miR-195 and the mean scores of BDCAF and BSAS in BD patients with vascular involvement (p>0.05) (as shown in **Table 2**).

**Table 2.** Correlation of the serum levels of miR-195 with clinical disease activity.

	**MiR-195**	
	*r*	***p***
BDCF	0.219	0.303
BSAS	0.150	0.485

MiR: MiRNA; BDCAF: Behçet’s Disease Current Activity Form; BSAS: Behçet’s Syndrome Activity Scale; All values are Spearman’s correlation coefficients (95% confidence intervals); p<0.05, statistically significant.

## DISCUSSION

This study found that BD patients exhibited higher plasma expression levels of miR-195, miR-424, miR-10b, miR-103a-3p, and miR-542-3p compared to healthy controls. In addition, BD patients with vascular involvement had higher serum miR-195 expression levels than those without vascular involvement. However, no correlation was observed between serum levels of miR-195 and clinical disease activity. To our knowledge, this is the first study to investigate the association of miR-195, miR-424, miR-10b, miR-103a-3p, and miR-542-3p with vascular involvement in BD patients.

miRNAs have been shown to play a significant role in regulating immune responses by controlling the differentiation of various immune cell subsets.^28^ For instance, Ding et al. demonstrated the role of miR-424 in Th17 cell proliferation.^29^ Interestingly, the infiltration of neutrophils and monocytes into inflamed tissues in BD has been linked to the reaction of T helper (Th)-17 cells. It has been suggested that Th17 cells, in concert with Th1 cells, are involved in the pathogenesis of BD through the interleukin (IL)-23-IL-17 axis.^30^ In this context, the study by Yuan et al. reported that miR-195 served as a regulator of CD4+ T cells.^31^ A published study demonstrated the complementary roles of CD4+ T cells in cytokine production and IL-17-related activity in patients with BD.^32^ Two other studies revealed the potent roles of miR-10b in neutrophil degradation; these studies demonstrated that miR-10b can act as a feedback loop targeting MAP3K7 to suppress IL-17A.^33,34^ Moreover, two published studies revealed that serum levels of miR-542-3p may contribute to apoptosis resistance, and serum levels of miR-103a-3p may be elevated in inflammatory disorders.^35,36^ Consistent with these findings, our study showed that BD patients had significantly higher plasma expression levels of these miRNAs than healthy controls. The present study also suggested that these miRNAs may play a role in the pathogenesis of BD. However, clinical characteristics, such as medical therapy, may affect the serum levels of these miRNAs. For example, studies have shown that colchicine, TNF-alpha inhibitors, and cyclosporin A affect the serum levels of miRNAs in BD patients.^37–40^ Although the medical treatments were not statistically different between BD groups, the serum levels of miR-195 were significantly different in BD patients with vascular involvement. Therefore, this result may indicate a link between vascular involvement and miR-195. However, additional studies with a larger sample size are required to confirm this association.

Although numerous studies have demonstrated a relationship between miR-195 and vascular complications, there are no studies investigating this relationship in BD patients with vascular involvements.^41,42^ Jin et al. reported that serum miR-195 significantly contributed to the development of deep vein thrombosis by regulating apoptosis in vascular endothelial cells and downregulating Bcl-2 in peripheral blood.^43^ Ma et al. suggested that miR-195 suppressed aortic aneurysms via the TNF-α/NF-kB and VEGF/PI3K/Akt pathway, thereby inhibiting inflammation.^44^ Two published studies reported that miR-195 knockdown reduced inflammatory cell infiltration and secretion of thrombosis-related factors.^45,46^ Moreover, PHLPP2, a downstream target of miR-195, is a direct regulator of Akt phosphorylation.^45^ Wang et al. showed that overexpression of miR-195 could reduce the expression of interleukins (IL-6, IL-8, and IL-1b), NK-kB, and p38 MAPK simultaneously. They also suggested that miR-195 could suppress proliferative vascular disease through NF-kB pathways and intracellular signalling cascades such as p38.^47^ Serum miR-195 level is a potential indicator for thrombosis developing after acute myocardial infarction and orthopaedic surgeries.^48,49^ Interestingly, in our study, vascular-Behçet patients had higher miR-195 expression levels than BD patients without vascular involvement. These results imply a potential relationship between miR-195 and vascular involvement in BD patients. Further investigations are needed to evaluate the sensitivity and specificity of miR-195 in predicting vascular involvement.

In recent years, an increasing number of studies in the literature have explored the relationship between miR-195 and various rheumatological diseases, in addition to BD. For instance, Cheng et al. suggested that urinary exosomal miR-195-5p could serve as a novel biomarker in Lupus nephritis.^50^ Similarly, Bolha et al. identified dysregulated miR-195 involved in the regulation of vascular smooth muscle cell phenotype and intimal hyperplasia in giant cell arteritis (GCA) arterial lesions, implying its potential use as diagnostic and prognostic biomarkers of GCA.^51^ Furthermore, miR-195 was recognized as a general indicator of SpA (Spondyloarthritis) in a published study.^52^ Additionally, a study reported that miR-195-5p regulates tight junction expression through Claudin-2 downregulation in Ulcerative Colitis.^53^ However, no associations were found between gingival crevicular fluid miR-195-5p and periodontitis in Rheumatoid Arthritis.^54^ Despite these findings, it is important to note that more studies are needed to confirm the relationship between BD and other rheumatic diseases. Further research in this area will contribute to a better understanding of the role of miR-195 in various rheumatological conditions. The present study has two major limitations. Firstly, a prospective study would be more appropriate to determine whether the observed values remained stable during the treatment of vascular complications. The cross-sectional design of our study limited its ability to show this. Secondly, the disease activity assessment scales used in our study evaluated other organ involvements rather than vascular activation. It would be more valuable to use a scale, such as the Birmingham Vasculitis Activity Score, that specifically evaluates vascular involvement when assessing disease activity.

## CONCLUSION

Our study found that serum levels of miR-195 were elevated in patients with vasculo-Behçet’s disease, indicating its potential role in the immunopathogenesis of this condition and possible association with vascular involvement. Further research is needed to elucidate the underlying mechanism.

## CONFLICT OF INTEREST

The authors declare no conflict of interest.
